# The role of pain expectancy and its confidence in placebo hypoalgesia and nocebo hyperalgesia

**DOI:** 10.1097/j.pain.0000000000003495

**Published:** 2025-01-09

**Authors:** Eleonora Maria Camerone, Giorgia Tosi, Daniele Romano

**Affiliations:** a Department of Psychology, University of Milano—Bicocca, Milano, Italy; b Nuffield Department of Clinical Neuroscience, University of Oxford Oxford, United Kingdom,; c NeuroMi—Milan Center for Neuroscience, Milan, Italy

**Keywords:** Expectation magnitude, Expectation precision, Placebo hypoalgesia, Nocebo hyperalgesia, Bayesian processing, Pain

## Abstract

Placebo hypoalgesia and nocebo hyperalgesia, which exemplify the impact of expectations on pain, have recently been conceptualised as Bayesian inferential processes, yet empirical evidence remains limited. Here, we explore whether these phenomena can be unified within the same Bayesian framework by testing the predictive role of expectations and their level of precision (ie, expectation confidence) on pain, with both predictors measured at the metacognitive level. Sixty healthy volunteers underwent a pain test (ie, 8 noxious electrical stimuli) before (Baseline) and after (T0, T1, T2) receiving a sham treatment associated with hypoalgesic (placebo), hyperalgesic (nocebo), or neutral (control) verbal suggestions, depending on group allocation. Trial-by-trial expectations, their precision, and perceived pain were measured. Skin conductance response (SCR) was also recorded as an autonomic response marker. Bayesian linear mixed models analyses revealed that, for both placebo and nocebo, pain was predicted by expectations alone and by their interaction with expectations precision. In addition, the discrepancy between expected and perceived pain was predicted by expectation precision, with greater alignment between expected and perceived pain when precision was higher. This suggests that both placebo and nocebo responses are well described from a Bayesian perspective. A main effect of time for SCR was observed, suggesting habituation to painful stimuli. Our data provide evidence indicating that both placebo hypoalgesia and nocebo hyperalgesia can be unified within the same Bayesian framework in which not only expectations but also their level of precision, both measured at the metacognitive level, are key determinants of the pain inferential process.

## Abstract 2

Supplemental Digital Content is Available in the Text.

Pain is predicted not just by expectations (ie, prior) but also by their level of confidence (ie, prior precision).

## 1. Introduction

Placebo hypoalgesia and nocebo hyperalgesia are striking examples of biased perception.^7,49^ Here, the belief of having received an analgesic or hyperalgesic treatment, despite receiving an inert one, leads to expectations of pain decrease or increase, which in turn reduce or enhance pain perception.^11,28,29^

Contemporary “Bayesian brain” theories of perception, which envision our brain as a prediction machine generating top-down predictions about expected sensory inputs, have been widely used in vision^18^ and audition^13^ and have recently entered the realm of pain perception.^1,7,25,30,49^ These accounts conceptualise pain as stemming from the integration between the sensory data (ie, noxious sensory input) and the prior prediction (ie, expectations), weighted by their relative precision (ie, the inverse variance of the probabilistic representation) such that the greater the precision of each source, the greater its influence on the percept.^3,7,21^ Accordingly, the greater the prior precision, the greater its influence on the interpretation of the incoming sensory input (ie, percept shifts towards the prior), offering an explanation for perceptual biases, including placebo hypoalgesia and nocebo hyperalgesia.^1,7,33,49^ Metacognitively, the “prior” refers to one's expectation, whereas its “precision” can be thought of as the confidence one has in that expectation.^33,50^

Expectations are renowned for affecting treatment outcomes in pain patients^17,19,24,34,35^ and enduring dysfunctional expectations appear crucial in the transition from acute to chronic pain.^27,37^ Thus, testing novel Bayesian approaches to better understand expectation effects on pain is of utmost importance and may identify new treatment targets like expectation confidence. Placebo hypoalgesia and nocebo hyperalgesia provide excellent models for investigating the role of Bayesian accounts in expectancy-driven pain modulation within a controlled experimental setting before directly testing these models on clinical populations.

Despite the agreement within the scientific community that placebo hypoalgesia and nocebo hyperalgesia are Bayesian phenomena,^1,7,33,49^ most of the evidence remains indirect. Some placebo^2,26,38^ and nocebo^15,16^ studies have modulated expectations precision to test its effect on pain modulation, leading to promising results. However, these studies were not aimed at testing Bayesian inferential processes, and since expectation precision was not measured, its successful modulation can only be assumed. Within the realm of placebo, only 2 studies,^1,22^ to our knowledge, directly tested the predictive value of expectations in a model of placebo hypoalgesia accounting for the interplay between the expectation and its precision level, supporting Bayesian accounts. No similar data are available for nocebo hyperalgesia.

Here, we investigate whether placebo hypoalgesia and nocebo hyperalgesia elicited by verbal suggestions can both be described under the same Bayesian framework, according to which the percept is influenced not only by the expectation but also by its level of precision. We hypothesise that pain will be predicted by the interaction between the expectation and its precision and that the greater the expectation precision, the smaller the mismatch between what is expected and what is perceived. Importantly, expectation precision was measured at the metacognitive level, as previously done in cue-based paradigms^6,30,36^ but never implemented in placebo or nocebo research.

## 2. Methods

### 2.1. Participants

Sixty-nine healthy volunteers were recruited from the University of Milano-Bicocca student population by advertising the experiment around the University and using the University Recruitment System. Nine participants were excluded because their pain threshold exceeded the electrical stimulation safety limit fixed at 50 mA, resulting in a final sample of 60 healthy adult volunteers.

Sample size was determined targeting a group (3) by session (2) interaction effect in a between-subjects analysis of variance (ANOVA) design. This interaction effect indicates that placebo and nocebo effects are successfully induced. The software g*Power^20^ was used to calculate a sensitivity Power Analysis setting alpha and Power at the standard values of 0.05 and 0.8. By fixing the sample size to 60 participants, our design is sensitive to effects as small as *f* = 0.2. Importantly, linear mixed models (LMM) were used for our analyses, a method for which there is no standardised sample size calculation method yet.^23^ However, because LMMs have greater statistical power than traditional statistical methods such as ANOVAs, we can be confident that a sample of 20 participants per group is sufficient to capture the expected effect.

Participants had no severe scars or hand dysmorphia nor a history of chronic pain, neurological, or psychiatric conditions. They were not taking psychiatric drugs or analgesic treatments with daily dosing. They were instructed to refrain from taking analgesic and anti-inflammatory medications, tea, or coffee for at least 12 hours before the experiment. Data collection started in October 2022 and ended in January 2023. Experimental procedures were conducted according to the policies and ethical principles of the Declaration of Helsinki. The study was approved by the Ethics Committee of the University of Milano-Bicocca (registration number: 0110454).

### 2.2. Procedure

**Figure 1.**
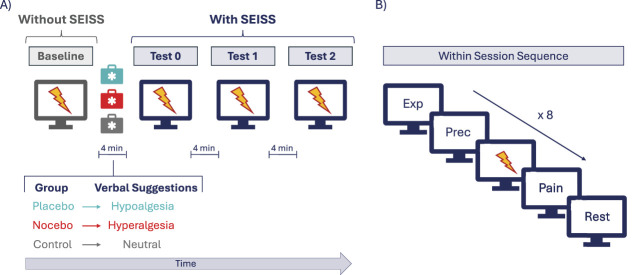
(A) Graphical representation of the experimental design illustrating the test session before (Baseline) and after (test 0, test 1, and test 2) the application of the sham treatment (ie, SEISS). After the Baseline, participants received verbal suggestions of hypoalgesia, hyperalgesia, or neutral, depending on group allocation. Test 0, test 1, and test 2 all ensued after the delivery of verbal suggestions, with participants receiving the sham treatment during these phases. (B) Baseline\Test sessions sequence including expectancy (ie, Exp) and confidence ratings (ie, Prec), noxious stimulus delivery (ie, lightning), and perceived pain rating (ie, Pain). This sequence is repeated 8 times for each test session.

Participants were recruited under the guise of a study investigating the hypoalgesic and hyperalgesic properties of a device called SEISS (“Stimolazione Elettromagnetica ad Intermittenza Sotto Soglia,” which translated in English becomes “Intermittent Subthreshold Electromagnetic Stimulation”). In truth, SEISS was an invented name for a magnetotherapy device, CombiGym Professional (“X Multi” model), which was never actively delivered as it was set on null parameters throughout the experiment (see Supplemental digital content SDC, Cover Story, http://links.lww.com/PAIN/C187). Participants sat on a comfortable chair in front of a computer screen used to display the pain and expectancy rating prompts (See Section: Expectancy, Pain Ratings, and Reaction Times) and to signal the incoming noxious stimulation during the experiment. With their dominant hand, participants held an external keyboard (Targus PAUK10 model) to give their ratings, while 4 electrodes were placed on participants' nondominant hand—ie, 2 electrodes positioned on the index and ring fingers to measure skin conductance response (SCR) (See Section: Skin Conductance Response) and 2 digital ring electrodes positioned on the middle one to induce noxious stimuli. Participants wore headphones to listen to white noise, isolating them from external noises. After consent, participants filled in demographic information and the STAI I questionnaire (See Section: Assessment of Psychological Characteristics). After the pain threshold was assessed (see SDC, Noxious Stimulation, http://links.lww.com/PAIN/C187), all participants underwent a familiarisation trial, a baseline and 3 test sessions (T0, T1, and T2). A 4-minute break was present between each test session. The familiarisation comprised 3 painful stimuli, whereas the baseline and the test sessions included 8 stimuli. All stimuli had a fixed intensity set at 2 times the current used to set the initial pain threshold (2T) (see SDC, Noxious Stimulation, http://links.lww.com/PAIN/C187) and an interstimulus interval of 25 seconds. Trial-by-trial expectancy and pain ratings were assessed in all sessions (See Section: Pain and Expectancy Ratings). During the 4-minute break between Baseline and T0, the initial 2 minutes were dedicated to informing participants about their group allocation and providing them with a leaflet detailing how SEISS worked (mirroring the information in the cover story). The remaining 2 minutes of the break were used to deliver specific verbal suggestions tailored to each group's allocation (See Section: Groups) (Fig. 1A). During the other rest periods between test sessions (ie, the 4-minute breaks between T0 and T1 and between T1 and T2), participants were instructed to wait patiently and relax without using their phones or similar devices. The sham treatment SEISS was administered during all the pain sessions after Baseline, ie, T0, T1, and T2. At the end of the experiment, participants filled in the remaining psychological questionnaires (See Section: Assessment of Psychological Characteristics). Participants were then debriefed, and a deception check was run by asking them whether they had understood that the study involved deception. Finally, participants were asked for their written consent to use their data in accordance with the true objective of the study. The total duration of the experiment was approximately 1h30.

### 2.3. Groups

Participants were randomised to 1 of 3 groups (N = 20 per group). All participants were administered the sham intervention SEISS, but they received hypoalgesic, hyperalgesic, or neutral verbal suggestions depending on group allocation:

(1) Placebo Group (P): “We will now repeat the pain test, but this time you will be under the pain-decreasing effect of the SEISS treatment.”
(2) Nocebo Group (N): “We will now repeat the pain test, but this time you will be under the pain-increasing effect of the SEISS treatment.”
(3) Control Group (C): “We will now repeat the pain test while you are receiving the sham SEISS treatment, which has no pain modulatory properties.”

The validity of these verbal suggestions has been demonstrated in Camerone et al.,^11^ in which the main difference was the presence of additional details on the temporality of the effects. Similar verbal suggestions are commonly used in the placebo and nocebo literature.^28^

### 2.4. Expectancy, pain ratings, and reaction times

Participants' expected pain intensity and confidence in their expectation were measured before each electrical stimulation by presenting the following questions on the computer screen in front of them: “How much pain do you expect from 0 (no pain) to 10 (unbearable pain)?” and “How confident are you about your prediction of the expected pain intensity from 0 (not confident at all) to 10 (extremely confident)?” A bar showing ratings from 0 to 10 was displayed underneath each question to help subjects give their evaluation. A lightning bolt symbol then appeared on the screen to prompt participants to the incoming noxious stimulation, delivered 5 seconds after the prompt onset. Next, a question asking how much pain they had experienced from 0 (no pain) to 10 (unbearable pain) was displayed with the 0-to-10 rating bar. After a 5-second rest period, the whole sequence started again and was repeated 8 times (Fig. 1B). Participants used an external keyboard (Targus PAUK10 model) to give ratings and had a 5-second window to respond to each question. Participants' reaction times (RTs) to give their pain and expectancy ratings were recorded.

### 2.5. Skin conductance response

Skin conductance response was recorded as a marker of autonomic arousal. We were interested in the peak-to-peak (PP.P-P) evoked response as a marker for pain intensity, as suggested in previous studies.^40,41^ Precisely, we focused on the peak-to-peak of the 5-second time window after the arrival of the electrical stimulus. Skin conductance response was measured using the BIOPAC MP150 system with the AcqKnowledge software. Electrodermal activity was captured using 2 electrodes applied to the index and ring fingers of the nondominant hand. The recording was stored for offline analysis. A high-pass filter set at 0.05 Hz was used to derive phasic skin conductance responses from the tonic level as suggested by Biopac Company, the gain parameter was set at 5 μS/V.

### 2.6. Assessment of psychological characteristics

Participants completed 6 questionnaires measuring psychological features associated with placebo and nocebo responsiveness. All questionnaires were administered in their Italian validated version: (1) *State-Trait Anxiety Inventory* (*I-II; STAI I-II*) to measure state and trait anxiety,^46^ (2) *Fear of Pain Questionnaire* (*FPQ-III*) to measure one's fear of pain,^32^ (3) *Pain Catastrophizing Scale* (*PCS*) to measure one's level of pain catastrophism,^48^ (4) *Life Oriented Test Revised* (*LOT-R*) to evaluate optimistic or pessimistic attitudes,^44^ (5) *Rosenberg Self Esteem* (*RSE*) measuring self-esteem,^42^ and (6) *General Self Efficacy* (*GSE*) to measure self-efficacy.^45^

### 2.7. Statistical analysis

Frequentist analyses for the demographic variables were performed using the Statistical Software Jasp (Version 0.16.3 for Apple). Bayesian linear mixed models (BLMM) were used for the remaining analyses, both for the preliminary and the main outcomes, using the R package *brms*, version 2.19.0.^8,9^ The script for the BLMM analyses, along with its corresponding data, is stored in the OSF Storage, accessible using the following link: https://osf.io/zp4cn/?view_only=236d7f792e65406fb05f397f9b30e765.

#### 2.7.1. Participants characteristics

To test for group differences in demographic variables, psychological questionnaire scores and deception check scores, the 3 groups were compared using ANOVA for interval level variables, or χ^2^ test for categorical variables. Group baseline differences for pain and expectancy ratings were tested using BLMMs (see SDC, http://links.lww.com/PAIN/C187, Group baseline differences analyses).

### 2.8. Preliminary analyses

Preliminary analyses were run to check whether the experimental manipulation we used (ie, verbal suggestions modulation) worked as expected, eliciting placebo hypoalgesia and nocebo hyperalgesia responses and influencing expectations coherently. Three separate BLMMs analyses investigated whether pain (ie, Pain), expectancy (ie, Expectation), and SCR (ie, P-P) were predicted by the experimental conditions. The variable ID (ie, the participants) was included as a random effect variable estimating both the random intercept and the random slope on all models' parameters.

#### 2.8.1. Were there placebo and nocebo effects?

To test whether verbal suggestions modulated pain (ie, elicited placebo hypoalgesia and nocebo hyperalgesia), a BLMM analysis was run, including groups (P, N, C) and session (Baseline, T0, T1, T2) as fixed effects and Pain as the dependent variable (DV). We used sequential contrasts. In this model, each level of session is compared with the following one (Baseline vs T0; T0 vs T1; T1 vs T2), allowing tracking of the updating over time. Weakly informative priors were set for the fixed effect regression coefficients (ie, *normal* (0,5)) and the intercept (ie, *normal* (5,1.5)). To rule out a potential role of trial, we tested whether placebo and nocebo effects still occurred when accounting for trial variability by running the same analysis but including trial as an additional random effect predictor in the model (see SDC, http://links.lww.com/PAIN/C187, Model including trial).

#### 2.8.2. Were expectations modulated?

To investigate whether verbal suggestions modulated expectations (ie, elicited expectations of hypoalgesia and hyperalgesia), a similar BLMM analysis was conducted, including groups (P, N, C) and session (Baseline, T0, T1, T2) as fixed effects and Expectation as the DV. By using sequential contrasts, each level of session is compared with the following one (Baseline vs T0; T0 vs T1; T1 vs T2). The distribution of Expectation did not follow a normal distribution but rather a cumulative distribution, which requires the data to be integers greater than 0. Accordingly, Expectation was transformed into Expectation.1 by shifting the values of 1 to have only positive values and avoiding 0 (ie, instead of having values starting from 0, they start from 1). Priors were set as in the previous analysis (ie, Fixed effect regression coefficients, *normal* (0,5); intercept prior, *normal* (5,1.5)).

#### 2.8.3. Were placebo analgesia and nocebo hyperalgesia responses mirrored in physiological changes?

To evaluate whether verbal suggestions had an impact on SCR, as a physiological correlate of pain perception, an additional BLMM analysis was conducted, including groups (P, N, C) and session (Baseline, T0, T1, T2) as fixed effects and P-P as the DV. Also in this case, by using sequential contrasts, each level of session is compared with the following one (Baseline vs T0; T0 vs T1; T1 vs T2). To obtain normally distributed data, the DV (ie, P-P) was calculated from the square root of PP.P-P., which was not normally distributed. Weakly informative priors (ie, *normal* (0,5)) were set for the fixed effect regression coefficients, whereas priors for the intercept were estimated by the *get_prior* function (ie, it assumes weakly informative priors).

### 2.9. Main analyses

The main analyses investigated whether placebo hypoalgesia and nocebo hyperalgesia (ie, perceptual biases) elicited by verbal suggestions modulation (as verified in the preliminary analyses) follow Bayesian inferential rules, according to which perceived pain results from the integration between the sensory data, the prior and their level of certainty. In our experiment, the sensory data were kept fixed by delivering electrical stimuli of fixed intensity, allowing us to exclude the input of sensory data from our predictive model. The prior is considered at the metacognitive level by measuring Expectation (ie, expectation of incoming pain), and its precision is quantified by measuring one's confidence in their expectation (ie, Precision). In these analyses, the Bayes factor (BF) was computed by comparing the target model (M_1_) with a new model (M_2_), which is identical to M_1_ except for the effect of interest that is excluded, allowing estimating the evidence favouring the inclusion of the effect of interest.

The first main analysis tested whether the Bayesian framework is a good model for the data by investigating if pain (ie, percept) is predicted by the interaction between the 2 prior dimensions: Expectation × Precision. A BLMM analysis was conducted, including Expectation, Precision, and their interaction as fixed effects and Pain as the DV. Weakly informative priors (ie, *normal* (0,5)) were set for the fixed effect regression coefficients and the intercept prior (ie, *normal* (5,1.5)). Priors for the random effects and model-specific parameters were estimated by the *get_prior* function (ie, it assumes weakly informative priors). The BF was computed by comparing M_1_ with M_2._ M_2_ was equal to M_1_ but without the effect of interest, which in this case is the interaction between Expectation and Precision, thus a model with just the main effects.

The second main analysis tested whether the data follow Bayes rules by looking at whether the prior precision (ie, Precision) predicts the discrepancy between what is expected and what is perceived. To this end, DeltaPain was computed as the absolute difference between the expectation and the pain (ie, DeltaPain = |Expectation − Pain|). The distribution of DeltaPain did not follow a normal distribution but rather a cumulative distribution, which requires the data to be integers greater than 0. Thus, DeltaPain was transformed into Delta.Pain.1 by shifting the values of 1 to have only positive values and avoiding 0 (ie, instead of having values starting from 0, they start from 1). A BLMM analysis was conducted, including Precision as the fixed effect and DeltaPain.1 as the DV. Weakly informative priors (ie, *normal* (0,5)) were set for the fixed effect regression coefficients. By contrast, priors for the intercept of the fixed effect, priors for the random effects, and model-specific parameters were estimated by the *get_prior* function (ie, it assumes weakly informative priors). The BF was computed by comparing M_1_ with M_2._ In this model, M_2_ is equal to the null model (M_0_), in which DeltaPain.1 only has the intercept.

## 3. Results

### 3.1. Participants characteristics

Analysis of variances comparing the scores of the psychological scales (ie, STAI I-II, FPQ-III, PCS, LOT-R, RSE, GSE) between groups did not reveal any significant differences (*Ps* > 0.05). In addition, the 3 groups did not differ significantly in age (*P* = 0.383). Chi-square tests of independence showed no significant association between gender and group allocation (*P* = 0.93) (Table S1, http://links.lww.com/PAIN/C187). Chi-square tests of independence did not reveal a significant association between deception check evaluation and group (*P* = 0.124). As reported in Table S1, http://links.lww.com/PAIN/C187, most participants in each group (C = 68.42%; *P* = 85%; N = 70%) indicated that they had not suspected any deception. Bayesian linear mixed models did not reveal differences between the 3 groups at baseline in terms of pain and expectancy ratings (see SDC, http://links.lww.com/PAIN/C187, Group baseline differences analyses).

### 3.2. Preliminary analyses

#### 3.2.1. Were there placebo and nocebo effects?

**Figure 2.**
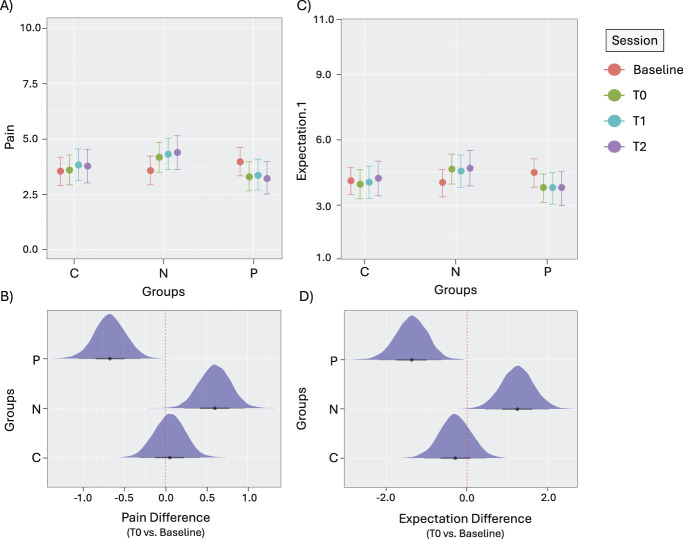
Panels (A and C): BLMM analyses including groups (P, N, C) and session (Baseline, T0, T1, and T2) as fixed effects and Pain (panel A) and Expectations.1 (panel C) as the DVs. For both plots (A and C), the x-axis is the variable Groups (C, N, P), and the variable Session is depicted in pink for Baseline, in green for T0, in blue for T1 and in purple for T2. The y-axis is the variable Pain in panel (A), and the variable Expectation.1 in panel (C). Error bars represent the 95% credible intervals. BLMM, Bayesian linear mixed model; DV, dependent variable. Panels (B and D): Distributions of the interaction effect between Group and Session at T0 (contrasted with baseline) for Pain (panel B) and for Expectations.1 (panel D). For both plots (B and D), the y-axis is the variable Groups (C, N, P). In panel B, the x-axis shows values for the distribution of the effect of T0 vs Baseline for Pain, and in panel (D) the x-axis shows values for the distribution of the effect of T0 vs Baseline for Expectations.1. Error bars represent the 95% credible intervals. From panel (B and D), it is clear that the distribution for the (C) group is around the 0 (ie, no-difference between T0 and baseline), whereas the distributions for the N and P groups are shifted towards positive and negative values, respectively, indicating pain/expectancy decrease and pain/expectancy increase at T0 compared with baseline.

This analysis factoring Group and Session as fixed effects, and Pain as the dependent variable indicates that there is a consistent group-by-session effect for the contrast Baseline vs T0 (Table 1A). We can be 95% confident that the difference in pain perception between N and C at T0 is higher (between 0.03 and 1.06) than in Baseline, indicating greater pain in the N at T0 compared with C (Figs. 2A and B). In addition, we can be 95% confident that the difference in pain perception between P and C at T0 is also greater (but in the opposite direction compared with N; between −1.25 and −0.20) than in Baseline, indicating decreased pain in the P at T0 compared with C (Figs. 2A and B). These results support the presence of hypoalgesic and hyperalgesic effects that are in line with the delivered verbal suggestions, indicating that our experimental manipulation (ie, verbal suggestion modulation) was successful at inducing placebo hypoalgesia and nocebo hyperalgesia. No other contrasts resulted to be likely different from 0 (ie, T0 vs T1; T1 vs T2), indicating that, once triggered, both placebo hypoalgesia and nocebo hyperalgesia remained stable over time (Table 1A).

**Table 1:** Population-level effects of the preliminary analyses, ie, *a*) Pain ∼ Group × Session and *b*) Expectation.1 ∼ Group × Session.

	Estimate	Est.Error	1-95% CI	u-95% CI	Rhat	Bulk_ESS	Tail_ESS
a) Pain ∼ Group × Session							
Intercept	3.69	0.33	3.02	4.34	1.01	771	1226
Groups N	0.42	0.46	−0.48	1.34	1.01	554	1377
Groups P	−0.23	0.46	−1.10	0.69	1.01	792	1647
Session T0	0.05	0.19	−0.31	0.42	1.00	1797	3220
Session T1	0.24	0.13	−0.01	0.48	1.00	2554	3643
Session T2	−0.06	0.15	−0.36	0.24	1.00	3019	4401
Groups N × Session T0	0.55	0.27	0.03	1.06	1.00	1986	4066
Groups P × Session T0	−0.73	0.27	−1.25	−0.20	1.00	1793	3395
Groups N × Session T1	−0.09	0.18	−0.44	0.25	1.00	2805	3560
Groups P × Session T1	−0.17	0.18	−0.53	0.18	1.00	2982	4760
Groups N × Session T2	0.13	0.21	−0.28	0.56	1.00	3228	4981
Groups P × Session T2	−0.08	0.22	−0.52	0.35	1.00	2890	4218
b) Expectation.1 ∼ Group × Session							
Intercept [1]	−5.40	0.71	−6.77	−4.01	1.00	813	1829
Intercept [2]	−3.12	0.68	−4.43	−1.75	1.00	769	1513
Intercept [3]	−1.25	0.68	−2.55	0.11	1.00	760	1625
Intercept [4]	0.84	0.68	−0.48	2.19	1.00	765	1437
Intercept [5]	2.73	0.69	1.42	4.10	1.00	778	1498
Intercept [6]	4.83	0.69	3.51	6.22	1.00	798	1569
Intercept [7]	6.89	0.70	5.58	8.28	1.00	827	1691
Intercept [8]	8.23	0.71	6.89	9.66	1.00	868	1818
Intercept [9]	9.42	0.73	8.03	10.91	1.00	922	2000
Intercept [10]	11.15	0.78	9.64	12.71	1.00	1071	2422
Groups N	0.79	1.01	−1.20	2.82	1.01	640	1234
Groups P	−0.24	1.01	−2.19	1.73	1.00	697	1436
Session T0	−0.30	0.39	−1.09	0.47	1.00	2628	4244
Session T1	0.18	0.27	−0.35	0.71	1.00	3273	4392
Session T2	0.35	0.29	−0.22	0.94	1.00	4856	5713
Groups N × Session T0	1.54	0.56	0.45	2.65	1.00	2600	3903
Groups P × Session T0	−1.09	0.54	−2.16	−0.05	1.00	2673	4336
Groups N × Session T1	−0.37	0.37	−1.10	0.35	1.00	2973	4721
Groups P × Session T1	−0.18	0.38	−0.93	0.56	1.00	2775	4669
Groups N × Session T2	−0.09	0.41	−0.91	0.72	1.00	5329	5625
Groups P × Session T2	−0.34	0.41	−1.15	0.45	1.00	4941	5668

For each parameter, Bulk_ESS and Tail_ESS are effective sample size measures, and Rhat is the potential scale reduction factor on split chains (at convergence, Rhat = 1).

CI, confidence interval; Est.Error, estimated error; N, nocebo group; P, placebo group; T0, test 0; T1, test 1; T2, test 2.

#### 3.2.2. Were expectations modulated?

This analysis factoring Group and Session as fixed effects, and Expectation.1 as the dependent variable indicates a strong and consistent group-by-session effect for the contrast Baseline vs T0 (Table 1B). We can be 95% confident that the difference in pain expectation magnitude between N and C at T0 is higher (between 0.45 and 2.65) than in Baseline, indicating greater expectation of pain increase in the N than in the C group at T0 (Figs. 2C and D). In addition, we can be 95% confident that the difference between P and C at T0 in pain expectation magnitude is greater (between −2.16 and −0.05) compared with Baseline (again, the difference is in the opposite direction compared with N), indicating greater expectation of pain decrease in the P compared with the C group at T0 (Figs. 2C and D). These findings support the successful modulation of pain expectations coherently with suggestions of hypoalgesia and hyperalgesia. No other contrast showed effects likely different from 0 (ie, T0 vs T1; T1 vs T2), indicating that as for pain also for expectations, once triggered, the effects remained stable over time (Table 1B).

#### 3.2.3. Were placebo analgesia and nocebo hyperalgesia responses mirrored in physiological changes?

This analysis factoring Group and Session as fixed effects and P-P as the dependent variable indicates a main effect of Session at T0 such that we can be 95% confident that P-P at T0 is lower (between −0.14 and −0.01) than at Baseline (Table S2, http://links.lww.com/PAIN/C187). For Session, no other contrast showed effects likely different from 0 (ie, T0 vs T1; T1 vs T2). These results suggest a habituation effect of the SCR response, occurring between Baseline and T0, and remaining stable after that (Table S2, http://links.lww.com/PAIN/C187). No Group-by-Session interaction effects emerged from this analysis indicating that our experimental manipulation did not seem to modulate this physiological parameter.

### 3.3. Main analyses

**Figure 3.**
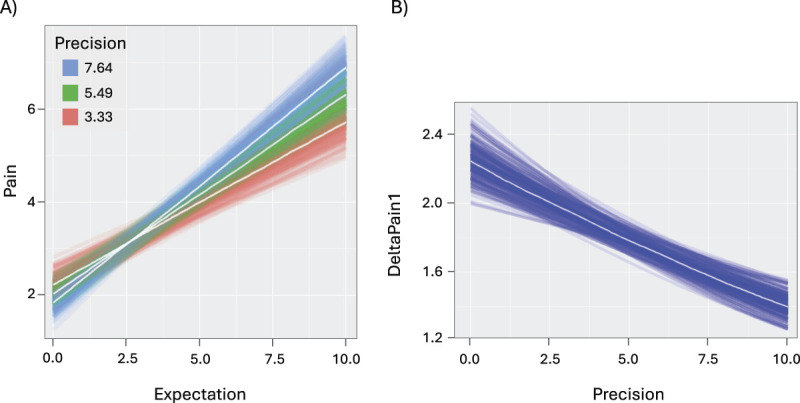
(A) BLMM analyses examining the predictive value of the interaction effect between Expectations (x-axis) and Precision (z-axis) on Pain (y-axis). Higher values of Precision (blue lines) correspond to a stronger relation between Expectation and Pain scores, whereas lower levels (red lines) are associated with a weaker relation. (B) BLMM analyses testing the predictive value of Precision (x-axis) on the discrepancy between expected and perceived pain computed as DeltaPain1 (y-axis). BLMM, Bayesian linear mixed model.

The first main analysis tested whether our data followed Bayes rules by assessing the predictive value of the interaction effect between expectations magnitude and precision on pain. This analysis, factoring Expectation and Precision as fixed effects and Pain as the dependent variable, indicates that there is a small but consistent Expectation × Precision interaction effect (Table 2A). We can be 95% confident that for each unit-increase in Precision, the relationship between Expectation and Pain changes of a value between 0.02 and 0.05. As depicted in Figure 3A, a higher Precision corresponds to a stronger relation between Expectation and Pain scores, providing evidence in support of the Bayesian account of pain modulation (ie, greater expectation precision leads to a percept that is closer to the prior). This analysis also revealed a main effect of Expectation (Table 2A) such that we can be 95% confident that for each unit-increase in Expectation, Pain changes of a value between 0.12 and 0.34, attesting to the strong relationship between expectations and perception. The BF was equal to 0.10648, indicating that the observed data are 1/0.10648 (=9.39) times more likely to occur under the model with only the main effect (M_2_) than under the model also including the interaction (M_1_). This indicates that the main effect of expectation magnitude is stronger than the interaction effect (as also evident from the magnitude of the effects: Estimate Expectation = 0.23; Estimate Expectation × Precision = 0.03, Table 2A). However, the interaction effect that we specifically tested for, despite being small, is different from 0, providing further insight into how expectations bias pain perception.

**Table 2:** Population-level effects of the preliminary analyses, ie, *a*) Pain ∼ Expectation × Precision and *b*) DeltaPain.1 ∼ Precision.

	Estimate	Est.Error	1-95% CI	u-95% CI	Rhat	Bulk_ESS	Tail_ESS
a) Pain ∼ Expectation × Precision							
Intercept	2.54	0.26	2.03	3.08	1.00	1944	3749
Expectation	0.23	0.06	0.12	0.34	1.00	2149	3901
Precision	−0.09	0.04	−0.16	−0.02	1.00	2276	4165
Expectation × Precision	0.03	0.01	0.02	0.05	1.00	2470	3939
b) DeltaPain.1 ∼ Precision							
Intercept [1]	−1.77	0.27	−2.31	−1.27	1.00	2827	4062
Intercept [2]	0.88	0.26	0.36	1.40	1.00	2906	4307
Intercept [3]	2.54	0.29	1.97	3.09	1.00	3271	4929
Intercept [4]	4.31	0.40	3.54	5.10	1.00	5076	6598
Intercept [5]	5.32	0.58	4.28	6.54	1.00	7133	5738
Intercept [6]	5.88	0.71	4.64	7.44	1.00	7396	5420
Intercept [7]	6.83	0.99	5.19	9.12	1.00	8727	5626
Intercept [8]	7.56	1.23	5.57	10.39	1.00	9512	5962
Precision	−0.24	0.04	−0.33	−0.15	1.00	2544	4210

For each parameter, Bulk_ESS and Tail_ESS are effective sample size measures, and Rhat is the potential scale reduction factor on split chains (at convergence, Rhat = 1).

CI, confidence interval; DeltaPain.1, DeltaPain (|Expectation − Pain|) with values shifted of 1; Est.Error, estimated error.

The second main analysis also tested the Bayesian account of pain modulation by investigating whether the expectation confidence predicted the match or mismatch between what is expected and what is perceived (DeltaPain.1). This analysis, factoring Precision as the fixed effect and DeltaPain.1 as the DV revealed a strong and consistent main effect of Precision, such that we can be 95% confident that for each unit-increase of Precision, DeltaPain1 changes of a value between −0.33 and −0.15 (Table 2B, Fig. 3B). As shown in Figure 3B, the higher the expectation precision (ie, Precision), the lower the mismatch between what is expected and what is perceived (DeltaPain1). Accordingly, the BF was equal to 2.504 × 10^12^, indicating with extreme evidence that data are more likely to occur under M_1_ than under M_0_. Altogether, these analyses indicate that our data are well described by a Bayesian perspective, according to which the higher the confidence in the prior, the more the percept aligns with the prior.

## 4. Discussion

The present study investigated whether placebo hypoalgesia and nocebo hyperalgesia elicited through verbal suggestions can be unified under the same Bayesian framework. Preliminary analysis indicated a decrease in pain perception for the placebo group and an increase for the nocebo group. Expectations were also modulated coherently with verbal suggestions. On these premises, we then tested whether the data followed Bayes' rules by examining the predictive role of the interaction between expectations and their precision on pain and by exploring the predictive power of expectation precision on the mismatch between what is expected and what is perceived (DeltaPain1). Innovatively, we conceptualised the prior (expectation) and its precision (confidence) at the metacognitive level, thus testing the Bayesian framework at a higher-order level compared with usual accounts of Bayesian conceptualisation in placebo research.^1^

Our first main analysis investigating the effect of expectation, precision, and their interaction on pain revealed a main effect of expectation, aligning with the extensive evidence supporting the significance of expectations in pain perception.^11,28,29^ This analysis also revealed a small but consistent interaction effect between expectation and precision, indicating that the relationship between expectation and pain changes as a function of expectation precision. Precisely, the association between the expectation and pain increases with the increase of precision, aligning with Bayesian rules suggesting that the greater the prior precision (expectation confidence), the more the percept (pain) will shift towards the prior (expectation). Bayesian factor computation comparing the reference model (expectation main effect) with the interaction model (expectation-by-precision) demonstrated that the simpler model exhibited greater predictive power, at odds with Bayesian principles anticipating greater predictive power for the interaction model.^7^ Despite the greater predictive power of the simpler model, we found an interaction effect different from 0 when testing the full model. Assessing the expectation-by-precision interaction effect is essential for directly evaluating the Bayesian probabilistic theory.^6^ Although previous research refrained from exploring this interaction because of insufficient statistical power,^6^ our study effectively tests this interaction by using LMMs, which enhance statistical power without necessitating an exceptionally large sample size.^31^ Our interaction results align with prior findings showing an expectancy-by-precision effect in placebo hypoalgesia. However, in this previous study, expectation precision was inferred from the data and the placebo effect was elicited using conditioning.^1^ We expand these results by (1) extending the validity of the Bayesian framework to a higher-order level by measuring expectations and their precision at the metacognitive level, (2) experimentally testing this framework for nocebo hyperalgesia for the first time, and (3) exploring its application when responses are elicited solely through verbal suggestions rather than conditioning. In relation to this last point, we highlight the importance of studying verbal suggestions and conditioning separately as they are different induction mechanisms with potentially different neural pathways—ie, both verbal suggestion and conditioning may have an effect on expectancy, but conditioning may also act using implicit learning.^4,39,43^ Computationally, although Milde et al.^33^ revealed that these induction procedures follow the same Bayesian integration when eliciting placebo effects, this has not yet been tested for nocebo hyperalgesia, warranting further investigation.

The second main analysis, testing the predictive power of expectation precision on the mismatch between what is expected and what is perceived (DeltaPain.1), strongly supports a Bayesian description of our data. A strong and consistent main effect of expectation precision was revealed, indicating a decrease in the mismatch between expected and perceived pain as the precision of one's expectations increased—ie, the greater the precision, conceptualised as confidence, the greater the alignment between the percept and the prior. This finding aligns with the smaller but present interaction effect of our previous analysis offering additional evidence towards a Bayesian conceptualisation of the data.

Although our 2 main analyses suggest that placebo hypoalgesia and nocebo hyperalgesia can be unified under the same Bayesian predictive model, the first analysis supports only a limited role of precision, whereas the second strongly favours the contribution of precision. This partial divergence could be explained by our experimental paradigm, where noxious stimuli were kept constant. The value of keeping the stimuli fixed, which allows assuming a fixed sensory parameter and isolating the expectation effect, comes at the cost of high predictability of the noxious stimuli, which could make expectancy ratings so accurate that they overshadow the smaller effect of precision. Since the first analysis tests the expectancy-by-precision interaction effect, the high accuracy of expectancy ratings is mirrored in the supremacy of the simpler model. Differently, the second main analysis isolates the effect of precision, thus overcoming the issue of expectancy accuracy overshadowing smaller effects by having precision as the only predictor and by looking at its effect on the expectation error (DeltaPain.1).

An exploratory analysis investigating whether expectation precision could be implicitly inferred from response time during expectancy rating (RT expectation) revealed that faster expectancy ratings predicted expectation precision, hinting that the time taken to rate the expectation could be a good implicit marker of self-reported expectation precision (see SDC, http://links.lww.com/PAIN/C187, “Exploratory Analysis”).

From a clinical standpoint, identifying expectation confidence as a factor in expectancy-driven pain modulation could offer a new target for treatment. However, it remains unclear if Bayesian processing applies to pain modulation in patients. Only 1 study has examined the predictive role of metacognitively measured expectations and their confidence in patients with back pain, finding an effect of expectations but not the interaction with precision,^34^ thus not supporting the translation of our findings to the clinical context. However, in this study, expectation and precision were only measured at 2 points (Baseline and T1), and because Bayesian inference involves continuous expectancy updating, future research recording ongoing expectancy ratings is required before drawing further conclusions.

The present study also contributes to the ongoing debate on the extinction behaviour of placebo hypoalgesia and nocebo hyperalgesia by showing that once triggered, both placebo and nocebo effects are maintained over time, aligning with previous findings using verbal suggestions^10–12^ and supporting their replicability and robustness. The evidence is more contradictory when conditioning is involved; although placebo hypoalgesia generally extinguishes more readily^2,14,16^ than nocebo hyperalgesia,^14,15^ the extinction or maintenance of these effects also depends on conditioning-specific features.^2,16^

In the context of these results, some considerations warrant acknowledgement. First, our study describes the interplay between pain expectancies, their level of confidence and pain ratings, but does not establish causality. The transition from the associative to the causal level can be achieved by experimentally manipulating the confidence attributed to the expectation and testing whether the effect of expectation on pain varies accordingly with expectation confidence. Some evidence shows that modulating the predictability of the prior, by changing either the conditioning^2^ or the verbal suggestions,^38^ affects the magnitude of placebo hypoalgesia (ie, greater predictability, greater hypoalgesia), whereas results remain contradictory for nocebo hyperalgesia (ie, greater predictability, greater hyperalgesia in one study^15^ but not in another study).^16^ None of these studies, however, measured the prior precision (expectation confidence), hindering the inference that changes in prior predictability necessarily led to shifts in its confidence, especially given recent arguments that uncertainty extends beyond unpredictability.^51^ Cue-based expectancy modulation studies are 1 step ahead, having modulated expectation certainty^6,36^ while also measuring it at the metacognitive level (confidence). Yet, these results remain controversial, aligning with the Bayesian prediction in one case (in Ref. 6, high-confidence expectations of receiving high-intensity noxious stimulus led to greater pain perception compared with low-confidence expectations) but not in another (in Ref. 36, lower expectation confidence was associated with greater expectancy-driven pain downregulation). Although these studies provide insight into the possible causal interplay between the prior confidence and the magnitude of the perceptual bias, none of them directly tested the Bayesian model by examining the predictive value of the expectancy-by-precision interaction, as done in this research. A second constraint of our study lies in the absence of a clearly defined physiological marker correlating with expectancy and pain responses. Skin conductance response peak-to-peak evoked response was chosen, based on previous evidence,^40,41^ as a physiological marker for pain anticipation and perception. However, a strong effect of time (ie, session at T0) was reported for this parameter, suggesting a habituation response potentially masking important effects. Future studies on Bayesian accounts of placebo/nocebo effects could use methods like electroencephalography to better explore neurophysiological correlates.^5,6,47^ An additional limitation is that this study was not preregistered.

In summary, our research offers compelling evidence supporting a unified model of placebo hypoalgesia and nocebo hyperalgesia, rooted in Bayesian probabilistic principles. Our findings indicate that pain perception is influenced not just by expectations but also by the confidence level assessed at the metacognitive level. The introduction of the Bayesian framework at the metacognitive level represents a groundbreaking advancement in placebo and nocebo research, opening avenues for further investigation. Importantly, our study introduces expectation confidence as a novel and yet-to-be-fully explored predictor, paving the way to exciting prospects for future research endeavours. From a clinical perspective, this novel predictor could be a potential target to explore the mechanisms underlying chronic pain onset and maintenance.

## Conflict of interest statement

The authors have no conflicts of interest to declare.

## Supplemental digital content

Supplemental digital content associated with this article can be found online at http://links.lww.com/PAIN/C187.

## Supplementary Material

SUPPLEMENTARY MATERIAL
