# Supplementary material for: The role of pain expectancy and its confidence in placebo hypoalgesia and nocebo hyperalgesia
Source: Pain. 2025 Jan 9;166(7):1577–86. doi: 10.1097/j.pain.0000000000003495 (PMC12168809; doi:10.1097/j.pain.0000000000003495)
Supplement: SUPPLEMENTARY MATERIAL [file jop-166-1577-s001.pdf]

Supplemental digital content (SDC)

The role of pain expectancy and its confidence in placebo hypoalgesia and nocebo hyperalgesia.

Eleonora Maria Camerone[1,2*], Giorgia Tosi[1], Daniele Romano[1,3]

Affiliations
[1] University of Milano – Bicocca, Department of Psychology
[2] University of Oxford – Nuffield Department of Clinical Neuroscience
[3] NeuroMi – Milan Center for Neuroscience

Corresponding Author: Eleonora Maria Camerone
Email: camerone.eleonora@gmail.com

This PDF file includes:

Supporting text
Group baseline differences analyses
Model including trial
Exploratory Analysis
Table S1
Table S2

Cover Story.

At the beginning of the experiment, participants were given the following information: "*This research aims to study the effects of an electromagnetic stimulation technique called SEISS, which stands for Intermittent Subthreshold Electromagnetic Stimulation, on the parasympathetic nervous system. SEISS can be used both to decrease and increase the perception of pain. How? Through a magnet that creates a magnetic field. In this case, we will apply the magnet on the arm, but it can also be applied to other parts of the body. By modulating specific parameters, we can create a polarising or depolarising magnetic field and consequently increase or decrease the perception of pain. While the effectiveness of SEISS on pain is well known, it is not clear whether it influences the parasympathetic nervous system or if this is bypassed. In this experiment, we investigate whether SEISS affects the parasympathetic system by measuring your Skin Conductance Response (SCR), a physiological correlate of the parasympathetic system's response which will be recorded using this* instrument [experimenter points toward the Biopac MP150 Systems]. *You will be assigned to one of three groups: either the group where we use SEISS to reduce pain, or the one where we use SEISS to increase pain, or the control group, in which SEISS is used in an inactive mode. We will tell you which group you have been assigned to during the experiment. Why do we tell you to which group you are assigned rather than keeping it blinded? We say this because we want*

to recreate a situation as similar as possible to the clinical context in which patients receiving a treatment know they are receiving it."

Noxious Stimulation.

Noxious stimuli were induced via electrical stimulation using two digital ring electrodes positioned 1 cm apart on the non-dominant hand's middle finger. Conductive gel was used. The stimuli were generated by Electrostimulator Digitimer DS7A/AH (High Voltage Constant Current Stimulator), which allows changing the intensity with an accuracy of 0.1 mA. The stimulation intensity was calibrated using an ascending staircase method as done by Camerone and colleagues [1]. To find the tactile threshold (t) and the pain threshold (T), three series of electrical stimuli were administered, starting at an intensity of 0.5 mA and increasing in steps of 0.5 mA. Each electrical stimulus had a pulse width of 500 µs and a maximum voltage of 300 V. Participants evaluated each stimulation on a Numerical Rating Scale (NRS) ranging from 0 to 10, where 0 indicated no pain, 1 represented the beginning of a painful feeling, 5 moderate pain and 10 unbearable pain. The calibration run was stopped when participants' ratings reached the pain threshold (i.e., stimulus intensity rated as 1). The pain threshold (T) was calculated by computing the average of the three pain threshold intensities. During the experiment, stimuli intensity was set at 2 times the current used to set the initial pain threshold (2T), except for during the conditioning manipulations.

Group baseline differences analyses.

Group baseline differences for pain and expectancy ratings were tested using BLMMs. Pain baseline differences were tested by running a model in which Pain is predicted by Group on the Baseline data points - i.e., BLMM analysis with Group (i.e., P,N,C) as fixed effect, Pain as the DV and the variable ID as random effect variable. Weakly informative priors were set for the fixed effect regression coefficients (i.e., *normal(0,5)*) and the intercept (i.e., *normal(5,1.5)*). We did not find evidence supporting an effect of Group on Pain at Baseline, suggesting that there are no relevant differences at Baseline between P and C and between N and C. We can be 95% confident that the difference between N and C is between -0.86 and 0.87, while the difference between P and C is between -0.48 and 1.28, indicating that the credible intervals are largely across 0. We have run a similar analysis to test Expectation baseline differences - i.e., BLMM analysis with Group (i.e., P,N,C) as fixed effect, Expectation as the DV and the variable ID as random effect variable. We can be 95% confident that the difference between N and C is between -0.94 and 0.74, while the difference between P and C is between -0.57 and 1.17, indicating that there is no effect of Group on Expectation at Baseline.

Model including trial.

To rule out a potential role of trial, we run an additional analysis to test whether placebo and nocebo effects still occur when accounting for trial variability. To this end, a BLMM analysis was run, including groups (P, N, C) and session (Baseline, T0, T1, T2) as fixed effects, Pain as the dependent variable (DV) and adding trial as an additional random effect predictor (in addition to ID). This analysis revealed that when controlling for trial variability, the strong and consistent group-by-session effect for the P group remains such that we can be 95% confident

that the difference in pain between P and C at T0 is higher (between -1.27 and -0.22) than in Baseline, indicating a greater decrease in pain in the P than in the C group at T0. Concerning the N group, our analysis revealed that, when accounting for trial variability, most of the effect was retained, with the estimate being very similar to the analysis without trial (i.e., it changes from 0.55 to 0.51) but with a slight effect on one of the tails of the credible intervals, which goes from -0.02 to 1.05. In this case, the difference in pain between N and C at T0 has a 95% probability of being a value between -0.02 to 1.05 compared to baseline, suggesting that pain in N is likely to be greater compared to the C group at T0, but with very small probability for the value to be 0. These results do not appear to greatly alter the interpretation of our model without trial, and overall, we do not see trial as a relevant biasing element for this specific research.

Exploratory Analysis.

An exploratory analysis was run to investigate whether the expectation precision (i.e., Precision) can be inferred implicitly from the time taken to give the expectancy rating (i.e., RT Expectation). To this end, a BLMM analysis was conducted, including RT Expectation as the fixed effect and Precision as the dependent variable (DV). Weakly informative priors (i.e., *normal(0,5)*) were set for the fixed effect regression coefficients and the intercept prior (i.e., *normal(5,1.5)*) was elicited from the data. Priors for the random effects and model-specific parameters were estimated by the *get_prior* function (i.e., it assumes weakly informative priors). The BF was computed by comparing M_1 to M_2. Also in this case, M_2 is equal to the null model (M_0) in which Precision only has the intercept.

This analysis, factoring RT Expectation as the fixed effect and Precision as the dependent variable (DV), detected a consistent effect of RT Expectation such that we can be 95% confident that for each unit-increase of RT Expectation, Precision changes of a value between -0.32 to -0.16, indicating that the shorter the RT to rate expectations (i.e., faster responses to rate the expectation of the incoming pain), the greater the reported confidence in such expectation, and vice-versa. Surprisingly, the estimated BF in favour of M_1 over M_0 was 1.308, indicating inconclusive evidence. This result is likely to be driven by the between-subjects high variability such that the null model with the random intercept is likely to explain our data as much as our model.

Altogether, this exploratory analysis, investigating whether expectation precision could be implicitly inferred from response time during expectancy rating (i.e., RT expectation), revealed that faster expectancy ratings are predicted by expectation precision. Thus, the time taken to rate the expectation seems to be a good implicit marker of self-reported expectation precision indicating that this parameter could be used as a proxy of expectation precision. However, before future studies can obviate the need to inquire about participants' confidence by using expectations reaction times as their proxy, further investigation is needed to test whether this association persists even when other sources of uncertainty interfere with the perception process (e.g., in our study, the fixed noxious stimuli allowed us to infer stable precision levels, which would not be the case with stimuli that vary trial-by- trial).

Table S1. Summary of the analyses (i.e., ANOVAs for interval level and chi-square tests for categorical variables) comparing the scores of the psychological scales (i.e., STAI I-II, FPQ-III, PCS, LOT-R, RSE, GSE), age, gender and deception check between the three groups.

	N	P	N	C	Statistics	p-value
**Age (mean ± SD)**	20	21±6.0	21±1.8	23±2.8	F(2,57)=0.98	0.383
**Gender (M/F)**	20	9/11	8/12	9/11	$X^2(2, N=60)=0.136$	0.934
**STAI I**	20	35±6.6	32±4.5	36±6.7	F(2,57)=1.87	0.163
**STAI II**	20	46±10.6	45±8.1	46±9.4	F(2,57)=0.13	0.877
**FPQ-III**	20	74±15.7	76±16.6	80±12.0	F(2,57)=0.81	0.451
**PCS**	20	20±10.0	17±8.6	21±10.2	F(2,57)=1.21	0.306
**LOT-R**	20	12±4.9	13±4.1	11±5.0	F(2,57)=1.07	0.350
**RSE**	20	18±4.5	18±2.9	18±5.2	F(2,57)=0.03	0.973
**GSE**	20	28±3.7	28±3.2	28±3.8	F(2,57)=0.13	0.881
**Deception Check (yes/no/maybe)**	20	0/17/3	1/14/5	4/13/2 (N=19)	$X^2(4, N=59)=7.243$	0.124

SD=Standard deviation; N=Sample size; M=Males; F=Females; P=Placebo Group; N= Nocebo Group; C=Control Group; STAI I/STAI II = State-Trait Anxiety Inventory (I-II); FPQ-III = Fear of Pain Questionnaire; PCS= Pain Catastrophizing Scale; LOT-R = Life Oriented Test Revised; RSE= Rosenberg Self Esteem; GSE= General Self Efficacy.

Table S2. Population-Level Effects of the preliminary analysis for SCR, i.e., P-P ~ Group *Session.

P-P ~ Group *Session	Estimate	Est.Error	1-95% CI	u-95%CI	Rhat	Bulk_ESS	Tail_ESS
**Intercept**	0.68	0.07	0.54	0.82	1.01	941	2014
**Groups N**	0.03	0.10	-0.17	0.23	1.01	914	1500
**Groups P**	0.01	0.10	-0.19	0.21	1.01	1020	1984
**Session T0**	-0.08	0.03	-0.14	-0.01	1.00	3452	4724
**Session T1**	-0.01	0.04	-0.09	0.07	1.00	2440	4048
**Session T2**	-0.02	0.02	-0.07	0.03	1.00	4935	5306
**Groups N* Session T0**	0.03	0.04	-0.06	0.11	1.00	3321	4866
**Groups P* Session T0**	0.06	0.04	-0.02	0.15	1.00	3552	5118
**Groups N* Session T1**	-0.04	0.06	-0.15	0.07	1.00	2604	4130
**Groups P* Session T1**	-0.02	0.06	-0.13	0.09	1.00	2669	3927
**Groups N* Session T2**	-0.01	0.04	-0.08	0.06	1.00	5789	5129
**Groups P* Session T2**	-0.05	0.03	-0.12	0.01	1.00	5162	5814

T0 = Test 0; T1= Test 1; T2= Test 2; P=Placebo Group; N= Nocebo Group; Est.Error =Estimated Error; CI=Confidence Interval. For each parameter, Bulk_ESS and Tail_ESS are effective sample size measures, and Rhat is the potential scale reduction factor on split chains (at convergence, Rhat = 1).

References.

[1] Camerone EM, Piedimonte A, Testa M, Wiech K, Vase L, Zamfira DA, Benedetti F, Carlino E. The effect of temporal information on placebo analgesia and nocebo hyperalgesia. Psychosom Med 2021;1:43–50.
